# The Potential of the Peptide Drug Semax and Its Derivative for Correcting Pathological Impairments in the Animal Model of Alzheimer’s Disease

**DOI:** 10.32607/actanaturae.27808

**Published:** 2025

**Authors:** A. I. Radchenko, E. V. Kuzubova, A. A. Apostol, V. A. Mitkevich, L. A. Andreeva, S. A. Limborska, Yu. V. Stepenko, V. S. Shmigerova, A. V. Solin, M. V. Korokin, M. V. Pokrovskii, N. F. Myasoedov, A. A. Makarov

**Affiliations:** Belgorod State National Research University, Belgorod, 308015 Russia; Engelhardt Institute of Molecular Biology, Russian Academy of Sciences, Moscow, 119991 Russia; National Research Center “Kurchatov Institute”, Moscow, 123182 Russia

**Keywords:** Alzheimer’s disease, peptide drug, behavioral testing, histological analysis, amyloidosis

## Abstract

Alzheimer’s disease, first described over a century ago, is currently among the most common neurodegenerative diseases whose significance is increasingly growing with the aging of populations. Throughout the entire period of its study, no remedies have been found that would be effective in treating – or at least significantly slowing – the pathological process, while being sufficiently safe. In this regard, significant attention is paid to the development and application of natural peptide drugs lacking side effects. The present study assessed the effect of the known neuroprotective peptide Semax and its derivative on the behavioral characteristics and development of amyloidosis in transgenic APPswe/PS1dE9/Blg mice acting as a model of Alzheimer’s disease. The open field, novel object recognition, and Barnes maze tests demonstrated that both Semax and its derivative improved cognitive functions in mice. Histological examination showed that these peptides reduced the number of amyloid inclusions in the cortex and hippocampus of the animals’ brains. These findings demonstrate the high potential of Semax and its derivatives when used to develop therapeutic and corrective strategies for Alzheimer’s disease.

## INTRODUCTION

Alzheimer’s disease (AD) is currently among the most prevalent neurodegenerative disorders in the elderly and senile populations [1, 2, 3, 4]. The progressive form of AD can be caused by cerebral disorders, intoxication, infection, and defects in the pulmonary and circulatory systems, leading to reduced oxygen supply to the brain, by nutrient and vitamin B12 deficiency, as well as by tumors [5, 6, 7, 8]. AD is the most common type of dementia and can be defined as a slowly progressive neurodegenerative disease characterized by the formation of senile plaques and neurofibrillary tangles via the accumulation of beta-amyloid peptide (Aβ) and tau protein within the most affected brain regions: the medial temporal lobe and neocortical structures [9, 10, 11].

The number of pharmaceuticals used to treat Alzheimer’s disease remains limited [12, 13]. Therefore, there is an ongoing need for novel compounds that would mitigate the cognitive impairment caused by disease progression [14, 15].

Animal models of AD play a crucial role in this research, as they make possible a detailed investigation of drug effects on key characteristics of the disease. The APPswe/PS1dE9/Blg (APP/PS1) transgenic mouse line, commonly used to study the mechanisms of AD and methods to correct them, is such a model [16].

Significant attention is currently directed toward developing drugs based on natural regulatory peptides, which are characterized by mild action and a lack of significant adverse effects [17]. Special attention has been focused on Semax, one of the wellknown and long-used peptide-based drugs containing the Met-Glu-His-Phe-Pro-Gly-Pro sequence. Semax is a hybrid molecule carrying an adrenocorticotropic hormone fragment, ACTH(4–7), and the Pro-Gly-Pro tripeptide, which affords increased resistance to peptidase activity. Semax does not exhibit any hormonal activity and is included in the Russian List of “Vital and Essential Drugs for Medical Application” (Appendix No. 1 to Decree No. 2406-r of the Government of the Russian Federation, dated October 12, 2019). It is used to treat neurological pathologies and stress conditions. Semax exhibits nootropic effects, stimulating learning, attention, and memory formation in animals and humans [18, 19, 20, 21, 22]. This very feature makes it a promising candidate for AD therapy. A preliminary trial of Semax in a limited cohort of AD patients showed that it can potentially be used to prevent and treat Alzheimer’s disease [23]. However, a further, more detailed investigation of the effects of the drug on various characteristics of AD is needed before its broader application [24, 25]. Furthermore, it is reasonable to study other derivatives of this peptide drug, whose architecture would incorporate structural features capable of improving the physiological properties of the potential therapeutic agent. This study employed a peptide derivative of Semax, with two amino acid substitutions. These substitutions (His-Phe to AspArg) resulted in the Glu-Asp-Arg sequence within its structure. Previous studies using cellular models of AD had indicated that the Glu-Asp-Arg tripeptide plays a positive role in improving the functional state of neurons [26].

The effects of Semax and its derivative, the Heptapeptide Met-Glu-Asp-Arg-Pro-Gly-Pro, on the behavior of APP/PS1 mice and the amyloid load in brain tissues were investigated in this study to assess the therapeutic potential of these peptides during the development of Alzheimer’s-type pathologic changes.

## EXPERIMENTAL

**Animals**

The experiments were conducted using 60 male APPswe/PS1dE9/Blg (APP/PS1) mice with a mixed C57Bl6/Chg genetic background and 20 male C57Bl6/Chg (wild-type, WT) mice. The housing conditions complied with the current sanitary regulations for the design, equipment, and maintenance of experimental biological clinics: ten animals per cage; temperature, 22°C; ad libitum access to water and forage; and a 12-h light cycle (from 8 a.m. to 8 p.m.). The laboratory animals with the Specific Pathogen-Free (SPF) status were procured from the Research Institute of Pharmacology of Living Systems at the Belgorod State University (Belgorod, Russian Federation). All the procedures were conducted in compliance with the Law of the Russian Federation “On the Protection of Animals from Cruel Treatment” dated June 24, 1998, the Good Laboratory Practice (GLP) regulations for preclinical studies in the Russian Federation (State Standards GOST 3 51000.3-96 and GOST R 53434-2009), and the EU Directive (86/609/EEC). All the stages of the study adhered to the Russell and Burch’s 3R principles.

**Synthesis of peptides and their characteristics**

Th e M e t-Glu-Asp-Arg-Pr o-Gly-Pr o p ep tid e (Heptapeptide) based on the adrenocorticotropic hormone fragment was synthesized by the conventional liquid-phase peptide chemistry approach using protected and free L-amino acids. The purity and identity of the synthesized compound were confirmed by high-performance liquid chromatography and mass spectrometry.

Semax, a synthetic peptide drug, an analog of ACTH_4-10_, which is entirely devoid of hormonal activity, was obtained according to the procedure described previously [4, 18, 25]. All the amino acids were L-stereoisomers.

**The formation of experimental groups**

**Fig. 1:**
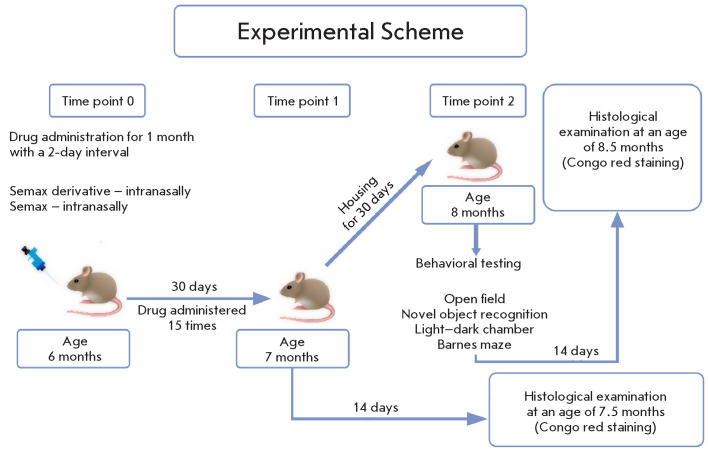
The experiment design in the study investigating the effects of Semax and the Heptapeptide on APP/PS1 mice

The mice were allocated into four study groups. The first group (the APP/PS1 group) was used as the positive control and comprised APP/PS1 mice with confirmed manifestations of Alzheimer’s-type pathology. The second group (the WT group) was the negative control and comprised wild-type animals. The third and fourth groups consisted of APP/PS1 mice intranasally administered either Semax (the Semax group) or its derivative (the Heptapeptide group) at a dose of 50 μg per kg, starting from the age of 6 months. The animals received the drug every other day during one month (a total of 15 doses). Next, the animals in the groups were divided into two subgroups. The first subgroup of animals in each group (10 animals) was allocated for histological analysis at the age of 7.5 months. The animals in the second subgroup underwent a one-month washout period during which they did not receive the drug. At an age of 8 months, the animals underwent behavioral testing during a 14-day period. The following tests were conducted: the open field test, the novel object recognition test, and the Barnes maze test. Once testing had been completed, histological examination of the animal’s brain sections was conducted (Fig. 1).

**Behavioral testing**

The open field test. This test allows one to assess the locomotor activity of, exploratory behavior, and anxiety in the animals. The animals were placed in an arena made of opaque plexiglass (50 × 50 cm base; wall height, 50 cm) (OpenScience, Russia). A single mouse was tested for 5 min under ambient lighting conditions of 35–40 lx. The following parameters were documented in the online test: the total number of movements; the total movement time (s); the total distance traveled (cm); the average speed of all the movements (cm/s); the resting time (s); the distance traveled in the peripheral zone (cm); the total time spent moving in the periphery zone (s); the distance traveled in the central zone (cm); the time spent in the central zone (s); and the number of center crossings. The EthoVision software (Version 16, Netherlands) was used for data recording and processing.

The novel object recognition test. This test was used to assess the animals’ cognitive functions, and memory in particular, by exploring the animals’ preference for exploring a novel object compared to a familiar one. The test is subdivided into three phases: the habituation, training, and testing phases. The open field test was conducted on day 1 of the novel object recognition test. On day 2 of the test, the animal was also placed in the arena for 5 min; two objects (toys) of the same color were placed in certain zones. On day 3, the animal was placed in the arena for 5 min again, one of the objects being replaced with a new one of a different color. The following parameters were recorded using the EthoVision software: the locomotor activity, the number of approaches made toward the novel and familiar objects, and the time spent near them. After data analysis, the preference index for the novel object was calculated using the formula:

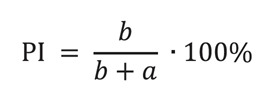



where a is the number of approaches to the old object and b is the number of approaches to the novel object.

The Barnes maze test. This test was used to study spatial learning and memory of the animals. The objective of the Barnes maze test was to let a mouse explore the space and memorize the location of the escape box using the configuration of distal visual cues placed around the testing area. The setup consisted of an arena 122 cm in diameter, with 40 holes 5 cm in diameter, one of them being the exit (the escape box). The distal visual cues were four blackand-white images with different figures and patterns, positioned in the cardinal directions (north, south, west, and east).

The Barnes maze test was carried out during five days: four days were intended for training and learning, and day 5 was the test day. During the first four days, the animals were placed in the arena for 3 min. After that, if the rodent had managed to find the escape box on its own, the box with the mouse inside was carefully transferred to its home cage. If the animal had failed to find the escape box independently, the experimenter gently guided it towards the box. Each rodent made four trials per training day with a 15-min interval. On day 5, the escape box was removed, and the hole was covered with a partition. The animal was placed in the arena for 5 min. The EthoVision software was used to measure the distance traveled, animal velocity, latency to find the target zone, and time spent near the former location of the escape box.

**Histological examination**

The animals were euthanized by cervical dislocation, and tissue specimens were prepared for analysis. The brain was dissected and fixed with Carnoy’s solution (six parts 96% ethanol, three parts chloroform, and one part glacial acetic acid) overnight. Tissue was dehydrated using a sequential series of ethanol solutions of increasing concentration: 75% solution, 1 h; 96% solution (I), 5 min; 96% solution (II), 45 min; 100% solution (I), 5 min; 100% (II). After incubation in a 100% ethanol–chloroform (1:1) mixture for 30 min and in chloroform (I) for 1 h, the specimens were left overnight in chloroform (II) and the tissues were then impregnated with paraffin (3 changes, 1 h each) at 60°C. Paraffin blocks were prepared using a Leica EG1160 tissue embedding center (Leica Biosystems). Paraffin sections (8 μm thick) were mounted onto polylysine-coated glass slides.

Five glass slides, each containing ten brain sections, were prepared from a 400 μm thick brain region; every fifth brain section was placed on a single glass slide. The sections were then deparaffinized in xylene for 20 min, rehydrated by sequential incubation in ethanol solutions (100% solution, 10 min; 95% solution, 5 min; and 50% solution, 5 min), followed by three washes with deionized water for 5 min. The sections were stained with a Congo red dye solution (0.5% Congo red in 50% ethanol) for 5 min, differentiated in a 0.2% KOH solution in 80% ethanol for 1 min, washed thrice with deionized water for 5 min, and embedded into the Immu-MountTM aqueous-based mountant (Thermo Scientific).

After brain fixation, dehydration, incubation, and paraffin embedding, tissue was sectioned and stained with Congo red dye. The total number of plaques across all the brain sections in the cortical and hippocampal regions was counted for each group throughout the study, and the arithmetic mean was calculated. Amyloid plaques were counted using the QuPath v0.5.1 software.

**Statistical analysis**

The descriptive statistics were employed for the statistical analysis of the data. All the behavioral testing data were characterized by parametric distribution by the Kruskal–Wallis H test. Two-way analysis of variance (two-way ANOVA) using generalized linear models (GLMs) was applied for intergroup comparisons in the Barnes maze test. Intergroup comparisons of changes in the histological variables were performed using the Kolmogorov–Smirnov test. Furthermore, the Šidák correction was used to control for the type I error rate during multiple hypothesis testing, which adjusts the significance threshold based on the number of planned comparisons. Differences were considered statistically significant at p < 0.05. The statistical analysis was conducted using the Statistica 10.0 software.

## RESULTS

**The effects of Semax and Heptapeptide on animal behavior**

**Fig. 2:**
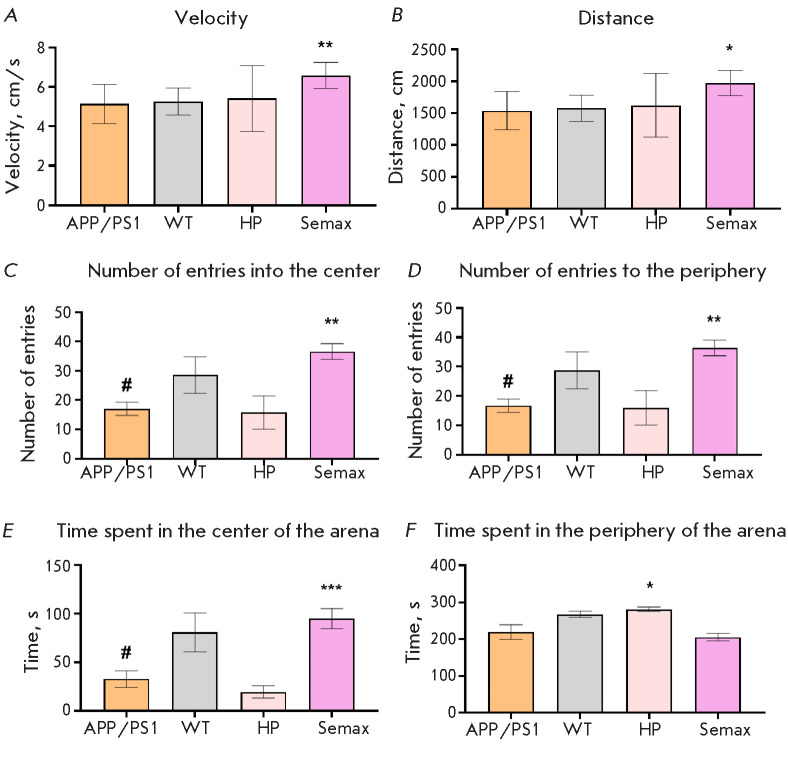
Results of the open field test. Velocity (A); total distance traveled (B); number of entries into the center zone (C); number of entries into the periphery zone (D); time spent in the center zone (E); and time spent in the peripheral zone (F). Here and thereafter: APP/PS1 (APPswe/PS1dE9/Blg transgenic mice), WT (wildtype animals), HP (APP/PS1 mice treated with Heptapeptide), Semax (APP/PS1 mice treated with Semax); * – p < 0.05, ** – p < 0.01, *** – p < 0.001, respectively (compared to the untreated APP/PS1 group); # – p > 0.05 (APP/PS1 mice compared to wild-type animals) (Kruskal–Wallis H test). Number of animals per group, n = 10

Mouse behavior in the open field test was evaluated at the first stage of the study (Fig. 2). A comparative analysis of the behavior of the animals in the WT and APP/PS1 groups revealed that the developing pathology in APP/PS1 mice statistically significantly reduced the number of entries into the center of the arena and entries into the periphery zone, as well as the time spent in the center of the arena. Administration of Semax prevented all these behavioral impairments in APP/PS1 animals. Furthermore, administration of Semax made the animals generally more active; their velocity and total distance traveled increased. Administration of the Heptapeptide statistically significantly increased the time spent in the periphery zone compared to the APP/PS1 animal group. However, the Heptapeptide exerted no significant effect on animal velocity, distance traveled, the number of entries into the periphery zone or center of the arena, or time spent in the center.

**Fig. 3:**
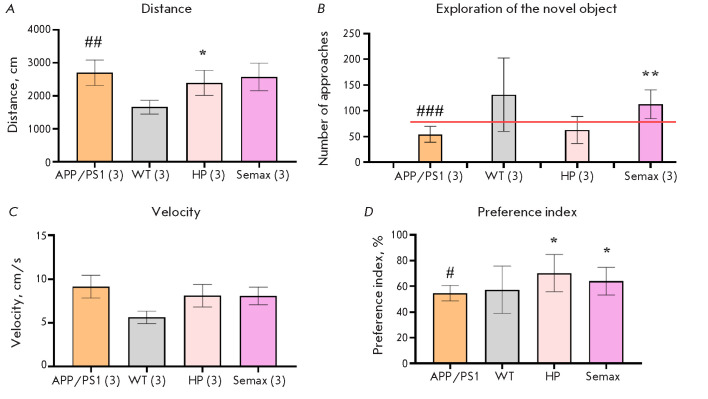
Results of the novel object recognition test on day 3. Total distance traveled (A); the number of approaches to the novel object on day 3 (B); velocity (C); discrimination index (D); * – p < 0.05, ** – p < 0.01, respectively (compared to the untreated APP/PS1 group); # – p > 0.05, ## – p < 0.01, ### – p < 0.001, respectively (APP/PS1 mice compared to wild-type animals) (Kruskal–Wallis H test). n = 10

The novel object recognition test was subsequently conducted (Fig. 3). On test day 3, the animals from the APP/PS1 group traveled a greater distance at a higher velocity compared to the animals in the WT group. Meanwhile, interest in exploring the novel object in APP/PS1 mice was significantly lower compared to that in wild-type mice. No differences in the preference index were observed for these two groups.

Administration of the Heptapeptide had no effect on the animals’ velocity, distance traveled, or exploration of the novel object. However, the preference index in these animals increased by almost 30% compared to that in the APP/PS1 mice. Administration of Semax significantly increased interest in the novel object and the preference index for the novel object in the animals.

**Fig. 4:**
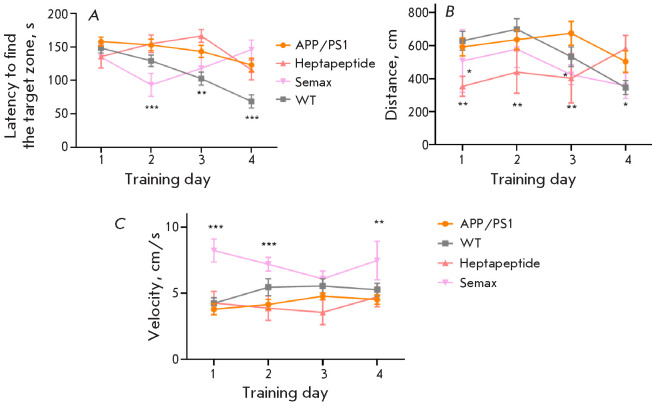
Results of the Barnes maze test (days 1–4). Latency to find the target zone (A); total distance traveled (B); velocity (C); * – p < 0.05, ** – p < 0.01, *** – p < 0.001, respectively (compared to the untreated APP/PS1 group), two-way ANOVA. n = 10

The Barnes maze test was then conducted. At the first stage of testing, the animals were trained during four days (Fig. 4). No significant differences in velocity and distance traveled were uncovered among all the studied groups, except for the animals receiving Semax. This drug significantly increased the velocity and reduced the distance traveled by the animals. On test days 2 and 3, the latency to find the target zone was significantly reduced in these animals; however, their performance deteriorated on test day 4.

**Fig. 5:**
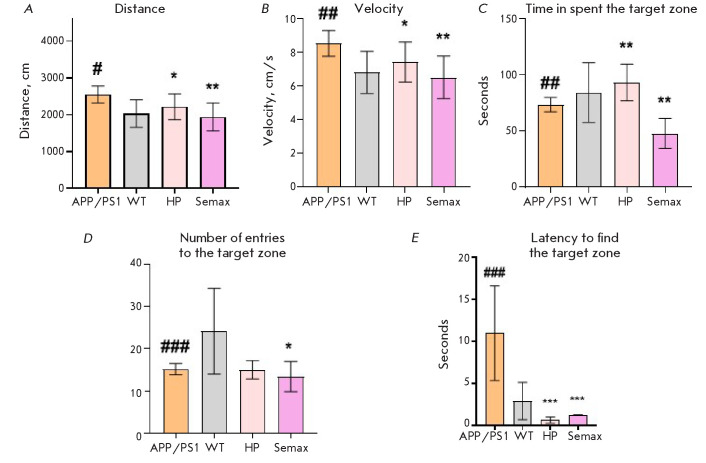
The results of the Barnes maze test (day 5). Total distance traveled (A); velocity (B); time spent in the target zone (C); number of entries into the target zone (D); latency to find the target zone (E); * – p < 0.05, ** – p < 0.01, *** – p < 0.001, respectively (compared to the untreated APP/PS1 group); # – p > 0.05, ## – p < 0.01, ### – p < 0.001, respectively (APP/PS1 mice compared to wild-type animals) (Kruskal–Wallis H test). n = 10

On day 5 of the Barnes maze test, the experimental trial was performed, where the animals were let go in the arena; the results are shown in Fig. 5. The APP/PS1 animals traveled a greater distance at a higher velocity and were slower to find the zone where the escape box had previously been located compared to the wild-type animals.

The administration of the Heptapeptide significantly reduced the distance traveled by the animals and their velocity, increased the time spent in the area where the escape box had previously been located, and reduced the latency to find the target zone compared to the APP/PS1 animal group. However, the Heptapeptide did not significantly affect the number of entries into the target zone. The drug Semax significantly reduced the velocity, distance traveled, time spent in the target zone, the number of entries into the target zone, and the latency to find the area where the escape box had previously been located. Therefore, both the Heptapeptide and Semax led to a notable correction in the behavioral parameters of the APP/PS1 animals to a level comparable to that in the wild-type animals.

**The results of histological studies**

The histological examination was performed to assess the effect of Semax and Heptapeptide on the development of amyloidosis in APP/PS1 mice. Experiments were carried out at two time points: the number of amyloid plaques in the animal brain was determined two weeks (in mice aged 7.5 months) and 1.5 months (in mice aged 8.5 months) after drug administration.

**Fig. 6:**
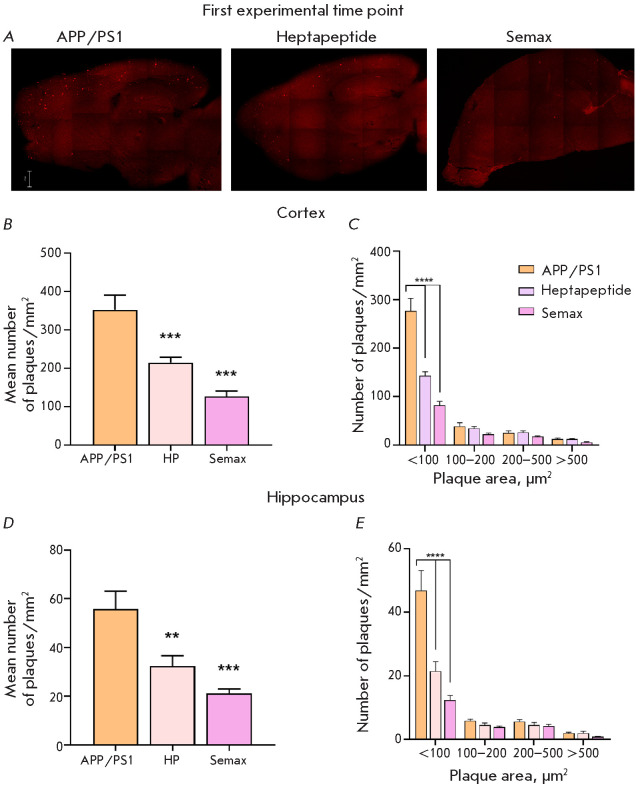
The results of the histological analysis of APP/PS1 mice at the age of 7.5 months. (A) Representative microphotographs of the brain sections from the control animals and mice administered Heptapeptide and Semax. Amyloid plaques are stained bright red. Scale bar: 500 µm. The mean number of amyloid plaques (B, D) and plaque size distribution (C, E) in the brains of APP/PS1 mice in the cortex (B, C) and hippocampus (D, E) at the first experimental time point. ** – p < 0.05, *** – p < 0.01, Kolmogorov– Smirnov test; **** – p < 0.0001, Šidák correction for multiple comparisons. n = 10

In the animals aged 7.5 months, therapy with Heptapeptide and Semax reduced the number of amyloid plaques in the cortical area by a factor of 1.6 and 2.8, respectively, compared to the untreated animals (Fig. 6). An analysis of the size distribution of amyloid plaques revealed that most of them were sized < 100 μm2 : those were the recently formed inclusions whose size would further increase with age. Heptapeptide and Semax significantly decreased the prevalence of this plaque population.

The same trend was observed when analyzing amyloid plaques in the hippocampal area (Fig. 6). The number of amyloid plaques in the Heptapeptide and Semax groups was 1.7-fold and 2.6-fold smaller than that in the APP/PS1 group, respectively. The druginduced reduction in the plaque count was most significant for the population of amyloid inclusions with an area of ≤ 100 μm^2^

**Fig. 7:**
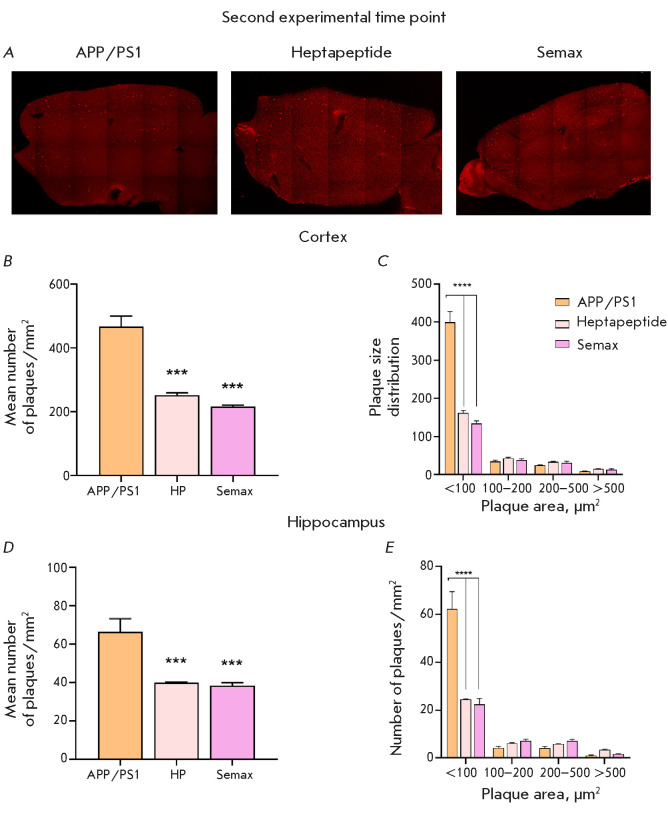
Results of the histological analysis of APP/PS1 mice at the age of 8.5 months. (A) Representative microphotographs of the brain sections from the control animals and mice administered with Heptapeptide and Semax. Amyloid plaques are stained bright red. The mean number of amyloid plaques (B, D) and plaque size distribution (C, E) in the brains of APP/PS1 mice in the cortex (B, C) and hippocampus (D, E) at the second experimental time point. ** – p < 0.05, *** – p < 0.01, Kolmogorov–Smirnov test; **** – p < 0.0001, Šidák correction for multiple comparisons. n = 10

In the APP/PS1 animals aged 8.5 months, the number of amyloid plaques was significantly increased both in the cortex and hippocampus (Fig. 7). Meanwhile, the protective effects of Heptapeptide and Semax were nearly identical to that observed for the 7.5-month-old animals. The numbers of amyloid plaques in the cortex of the mice in the Heptapeptide and Semax groups were 1.8-fold and 2.2-fold smaller than that in the APP/PS1 group. The number of plaques in the hippocampus of the mice in the Heptapeptide group was 1.6-fold smaller compared to mice in the APP/PS1 group, while the animals in the Semax group had 1.7 times fewer plaques. A size distribution analysis of amyloid plaques revealed that the largest reduction in number was observed for plaques sized up to 100 μm^2^ in mice of both ages (7.5 and 8.5 months) that had received the drugs.

## DISCUSSION

The peptide Semax, which exhibits neuroprotective and nootropic properties, is a long-acting memory enhancer [17]. The proposed modifications to Semax, yielding the Heptapeptide, are expected to enhance the effect of the peptide on the key pathological features of AD.

Testing of animal behavior revealed that APP/PS1 mice had significantly impaired behavioral and cognitive characteristics compared to those of wild-type mice. After a course of peptide drugs, many of these functions were restored, either completely or partially. Semax exhibited a significant favorable effect in the open field and novel object recognition tests. In the Barnes maze test, the Heptapeptide improved certain behavioral parameters of APP/PS1 mice to a level comparable to that of wild-type animals. Semax had a positive effect on an even greater number of parameters in this test.

Hence, a number of behavioral and cognitive characteristics in animals with a Alzheimer’s-type pathology showed improvement one month after the course of peptide drugs.

The most significant data were collected through the histological examination of animals’ brains. Amyloid plaques were detected in the cerebral cortex and hippocampus two weeks after the administration of the peptide during one month. At this stage, the mean number of amyloid plaques in the APP/PS1 group was > 350 per mm2 , while this number decreased 1.6-fold and 2.8-fold in the Heptapeptide and Semax groups, respectively. The peptides primarily reduced the number of small plaques (sized < 100 μm^2^ ), which is indication that they inhibit the formation of new plaques. As expected, the mean plaque number in the hippocampal region was smaller ( < 50 per mm2 ). In APP/PS1 mice, the peptides also reduced the number of plaques (1.7-fold for the Heptapeptide group and 2.6-fold for the Semax group). Therefore, at this stage, course therapy with either peptide significantly reduced the formation of amyloid plaques in both brain regions, Semax being the more efficient of these two peptides.

The next histological examination stage was conducted 1.5 months after drug administration using animal brain specimens. During this period, the mean number of plaques (per mm2 ) in the cerebral cortex of APP/PS1 animals had exceeded 400. In the Heptapeptide and Semax groups, this value increased only slightly, remaining significantly lower (1.8- and 2.2-fold, respectively). Similar findings were obtained for the hippocampal specimens. Hence, both peptides decreased the number of amyloid inclusions within tissues, and this effect persisted for 1.5 months postadministration. The effect of these peptides may potentially be based on an important feature of many peptides: their ability to allosterically interact with various receptors, thus altering their impact on controlled signaling pathways [27].

The previously proposed concept of amyloid matrices relies on a long-term interplay between modified beta-amyloid variants and partner proteins, including the alpha-4 nicotinic acetylcholine receptor. The resulting complexes can act as seeds for pathological aggregation of intact beta-amyloid molecules to induce the formation of amyloid plaques [28, 29]. Hence, it is fair to hypothesize that both of the studied peptides bind allosterically to receptors, including acetylcholine ones, and alter their configuration, thus either fully preventing or substantially reducing the degree to which they bind to the modified form of beta-amyloid. In this case, this particular amyloid plaque formation pathway can be inhibited by the peptides under study; this inhibitory effect persists for more than a month following the treatment course.

Our findings demonstrate that intranasal administration of Semax or Heptapeptide improves the cognitive function in the mouse model of Alzheimer’s disease. Both Semax and Heptapeptide significantly reduce the amyloid load in the animal brain. These data prove that Semax and its derivatives are promising for developing therapeutic and corrective strategies for Alzheimer’s disease.

## Abbreviations

AD: Alzheimer’s disease
Aβ: beta-amyloid peptide
APP/PS1: APPswe/PS1dE9 transgenic mice
ACTH(4-7): adrenocorticotropic hormone fragment (4-7)
Heptapeptide: Met-Glu-Asp-Arg-Pro-GlyPro peptide
WT: Wild type, C57BL/6 mice
